# The Role of Left Ventricular Outflow Tract Peak Velocity Measurement in Patients With Sepsis and Septic Shock

**DOI:** 10.7759/cureus.26840

**Published:** 2022-07-14

**Authors:** Mustafa Emin Serin, Yunus Emre Ozluer, Mehmet Kıy, Mucahit Avcil

**Affiliations:** 1 Emergency Medicine, Nevsehir State Hospital, Nevsehir, TUR; 2 Emergency Medicine, Adnan Menderes University, Aydin, TUR; 3 Emergency Medicine, Biga State Hospital, Çanakkale, TUR

**Keywords:** ultrasonography, critical care, septic shock, sepsis, left ventricular outflow tract

## Abstract

Aim

To determine whether left ventricular outflow tract peak velocity is useful for the prediction of mortality in the early phase of sepsis or septic shock.

Materials and methods

Patients who were hospitalized in the emergency intensive care unit (ED-ICU) with the diagnosis of sepsis or septic shock were consecutively enrolled into two groups (sepsis and septic shock groups) between January 2020 to February 2021. Patients who are pregnant and ≤18 years old were excluded. Demographics, vital parameters, the presence of mechanical ventilation, and vasopressor/inotropic support with the doses of the drugs used were recorded. Ultrasonographic measurements included bedside caval indexes and left ventricular outflow tract (LVOT) peak velocity measurements. The primary outcome was in-hospital and 28th-day mortality.

Results

A total of 116 patients with a median age of 72.5 (27 to 96) years were enrolled. Sixty-eight (58.6%) patients were male. According to a receiver operating characteristic (ROC) curve analysis, 75 cm/s was determined as a cut-off value to determine the efficacy of LVOT peak velocity measurement for discriminating septic shock from sepsis and predicting 28-day and in-hospital mortality. The patients were then regrouped as 54 (46.5%) patients in low and 62 (53.5%) patients in high-velocity groups according to the cut-off value. Both in-hospital and 28th-day mortality rates were significantly different between these groups (p<0.001).

Conclusion

Left ventricular outflow tract peak velocity measurement may be a useful adjunct for the prediction of mortality in septic patients. Vasopressors and volume status of the patient do not affect LVOT peak velocity measurements.

## Introduction

Sepsis continues to be one of the most prominent health problems with high rates of mortality [1]. Sepsis is defined as the state of life-threatening multiple organ dysfunction due to a disorganized host reaction to the cause of the infection [2]. However, septic shock defines the presence of a need for vasopressors to preserve mean arterial pressure (MAP) over 65 mmHg despite adequate fluid resuscitation and lactate > 2 mmol/L in the absence of hypovolemia [2]. Both situations mandate prompt recognition and early resuscitation. One of the main treatment strategies in sepsis and septic shock is intravenous fluid resuscitation. However, monitoring this process is essential to prevent the deleterious effects of volume overloading. Recent Surviving Sepsis campaign guidelines recommend using dynamic measures to guide fluid resuscitation over static measures [3].

A recently described method for the estimation of volume status in sepsis is the assessment of the obstruction of the left ventricular outflow tract (LVOT) by measuring the peak velocity of LVOT [4]. Although it is mainly associated with hypertrophic cardiomyopathy, it can also occur in sepsis and hypovolemia and is associated with the infusion of catecholamines and mechanical ventilatory support [5]. However, the data on this topic remains controversial. In our study, we aimed to investigate whether bedside LVOT peak velocity measurement gives different values in septic shock patients compared to sepsis patients. We also investigated the predictive value of LVOT peak velocity in predicting ICU mortality in these patients and the relationship between LVOT peak velocity and vasopressor drug administration.

## Materials and methods

Our study was conducted in the emergency department (ED) of a tertiary care center. Before starting the study, approval was obtained from the institution's ethics committee (approval no. 2019/111). Patients who were hospitalized from the ED to our emergency intensive care unit (ED-ICU) with the diagnosis of sepsis or septic shock between January 2020 to February 2021 were included in our study. Written and verbal informed consent was obtained from conscious patients, and from the relatives or legal representatives of the unconscious patients. Patients under the age of 18, who were pregnant, who had a known history of cardiac surgery, and whose consent could not be obtained were excluded from the study.

An a priori power analysis was conducted using G*Power3 to test the difference between two independent group means using a two-tailed test, a medium effect size (d=.50), and an alpha of .05. Results showed that a total sample of 116 participants with two equal-sized groups of n=58 was required to achieve a power of .80 [6]. A total of 116 patients, 58 of whom were diagnosed with sepsis and 58 with septic shock, were included in the study. We consecutively enrolled patients in both groups, and once one group was completed, patients from the other group were continued to be recruited. When diagnosing sepsis, according to the Sepsis-3 consensus diagnostic criteria published in 2016, the condition of obtaining two or more points from the sequential organ failure assessment (SOFA) score was sought in the presence of evidence or suspicion of infection. In the diagnosis of septic shock, according to the same consensus diagnostic criteria, vasopressor/inotropic support was required to keep MAP 65 mmHg and above, or the lactate level was above 2 mmol/L despite fluid resuscitation.

Demographic data included age, gender, comorbid disease status, diagnosis (sepsis or septic shock), and source of infection. Vital signs such as heart rate, systolic blood pressure, diastolic blood pressure, and MAP were also recorded. Furthermore, to investigate the severity of the disease SOFA and simplified acute physiology score 2 (SAPS2) scores, the presence of mechanical ventilation and vasopressor/inotropic support with the doses of the drugs used were recorded.

Left ventricular outflow tract peak velocity measurements were performed using a 2.5 to 3.5 MHz frequency sector probe in the supine position from the apical five-chamber window, and the subcostal window if images could not be obtained from these two windows. Once the appropriate view was obtained, we measured LVOT peak velocity with continuous wave (CW) spectral Doppler mode by placing the cursor onto the LVOT. Inferior vena cava (IVC) diameter measurement was made by measuring the maximum and minimum diameters with a convex probe with a frequency of 3 to 3.5 MHz. After the measurements, the caval index (collapsibility index = IVCmax-IVCmin/IVCmax) in patients with spontaneous breathing and the distensibility index (dIVC = IVCmax-IVCmin/IVCmin) in patients receiving mechanical ventilator support was calculated, and recorded. Patients with a collapsibility index of more than 50% and a distensibility index of more than 18% were considered to be having a fluid deficit. All ultrasonographic measurements were completed using Hitachi Aloka Prosound Alpha 6 (Hitachi Aloka Medical Systems, Tokyo, Japan). The outcomes including ICU mortality, length of stay, in-hospital mortality and modified Rankin scale on the 28th day were recorded.

Research data were analyzed using Statistical Package for Social Sciences version 20.0 (IBM Corp., Armonk, NY, USA) and Jamovi for Windows 1.6.13 programs. Variables within normal distribution were expressed as mean ± standard deviation (SD), and variables not within normal distribution were expressed as the median (min-max) values. Chi-square test was used in the analysis of categorical variables, Student's t-test in the analysis of numerical variables with normal distribution in independent groups, and the Mann-Whitney U for the analysis of numerical variables that did not fit the normal distribution. Relative risk analysis was performed to examine the effect of risk factors on mortality and morbidity. A cut-off value was determined by performing receiver operating characteristic (ROC) curve analysis to calculate the sensitivity and specificity, positive and negative likelihood ratio (LR), and the positive and negative predictive value of the LVOT peak velocity value in predicting the presence of septic shock and mortality. Kaplan-Meier survival analysis was used to determine the predictive effect of LVOT peak velocity on mortality. A multivariate Cox regression analysis was used to determine the factors affecting in-hospital and 28-day mortality. For statistical significance, the condition of determining p<0.05 was sought.

## Results

We enrolled 116 patients. Sixty-eight (58.6%) of them were male and the median age of the patients was 72.5 (27 to 96) years. Out of the total number of patients, 58 (50%) had sepsis, and 58 (50%) had septic shock. There were no significant differences in terms of sex and age among patient groups. Table 1 shows both the demographic and clinical features of the patient groups.

**Table 1:** Demographic and clinical features of the patient groups *According to the bedside measurements of inferior vena cava with ultrasound BP: Blood pressure, MAP: Mean arterial pressure, SOFA: Sequential organ failure assessment, SAPS: Simplified acute physiology score, LVOT: Left ventricle outflow tract, MV: Mechanical ventilation, ICU: Intensive care unit, mRS: Modified Rankin scale

	Sepsis (n=58)	Septic Shock (n=58)	p-value
Age (years), Median (min-max)	72.5 (35-91)	72 (27-96)	0.691
Male, n (%)	33 (56.9)	35 (60.3)	0.376
Presence of Comorbidities, n (%)	45 (77.6)	13 (22.4)	0.546
Heart Rate (bpm) ± SD	103.7±20.6	111.8±20.6	0.036
Systolic BP (mmHg) ± SD	106.7 ± 20.9	88.9 ± 23	<0.0001
Diastolic BP (mmHg) ± SD	63.6 ± 12.9	53.6 ± 12.6	<0.0001
MAP (mmHg) ± SD	77.3 ± 14.2	65.0 ± 14.9	<0.0001
Lactate (mmol/L), median (min-max)	2.8 (0.6-19)	3.6 (0.5-26)	0.028
SOFA Score, median (min-max)	5 (2-13)	8 (4-14)	<0.001
SAPS2 Score, median (min-max)	38.5 (15-65)	52 (13-76)	<0.001
LVOT Peak Velocity (cm/sec) ± SD	71.5 ± 16.4	87.3 ± 18.3	<0.001
Presence of hypovolemia*, n (%)	32 (55.2)	36 (62.1)	0.451
MV support, n (%)	7 (12.1)	23 (39.7)	0.010
ICU stay (days), median (min-max)	7 (1-99)	5.5 (1-63)	0.879
28-day mortality, n (%)	27 (46.6)	38 (65.5)	0.040
Overall ICU mortality, n (%)	31 (53.4)	42 (72.4)	0.034
28-day mRS, median (min-max)	5 (1-6)	6 (3-6)	0.009

Sixty-eight (58.6%) of the patients had at least one comorbid disease as seen above in Table 1. The prevalences of comorbidities in our study population were as follows: diabetes mellitus 21.6% (n=25), hypertension 29.3% (n=34), chronic renal failure 12.1% (n=14), chronic obstructive pulmonary disease 12.9% (n= 15), diseases regarding central nervous system including previous stroke, Alzheimer’s disease, and dementia 14.7% (n=17), and congestive heart failure 7.8% (n=9).

The most common source of infection was pneumonia in 34 (29.3%) patients. This was followed by intraabdominal infections (cholecystitis, cholangitis, intraabdominal abscess, intestinal perforation, peritonitis, etc.) in 24 (20.7%), urinary tract infections in 21 (18.1%), soft-tissue infections (necrotizing fasciitis, cellulitis, etc.) in 12 (10.3%), and other sources of infections such as gastroenteritis, febrile neutropenia, and fever of unknown etiology in 25 patients (21.6%). There was no statistically significant difference between sepsis and septic shock groups regarding the source of infection (p>0.05). 

Vasopressor support was initiated in 56 (48.3%) patients. With a median dose of 0.15 (0.01-1.70) µgr/kg/min, noradrenaline was the only agent used in this group of patients. The LVOT peak velocity was measured median of 90 (42-128) cm/s in patients who had noradrenaline infusion, and a median of 67 (41-125) cm/s in those who did not (p<0,0001). According to logistic regression analysis, administration of vasopressors was not found to be significantly affecting LVOT peak velocity measurements (B=1.006, SE=1.446, p=0.487).

The median length of stay in the ICU was six (1 to 99) days. Overall, seventy-three (62.9%) patients died, and 43 (37.1%) patients survived. The LVOT peak velocity was 69 ± 18.3 cm/s in patients who survived, and 85.5 ± 16.8 cm/s who did not (p<0,001). Furthermore, the LVOT peak velocity was a median of 65 (41-125) cm/s in 51 (43.9%) patients who survived 28 days, and a median of 89 (53-128) cm/s in 65 (56.1%) patients who did not (p<0,001).

**Figure 1:**
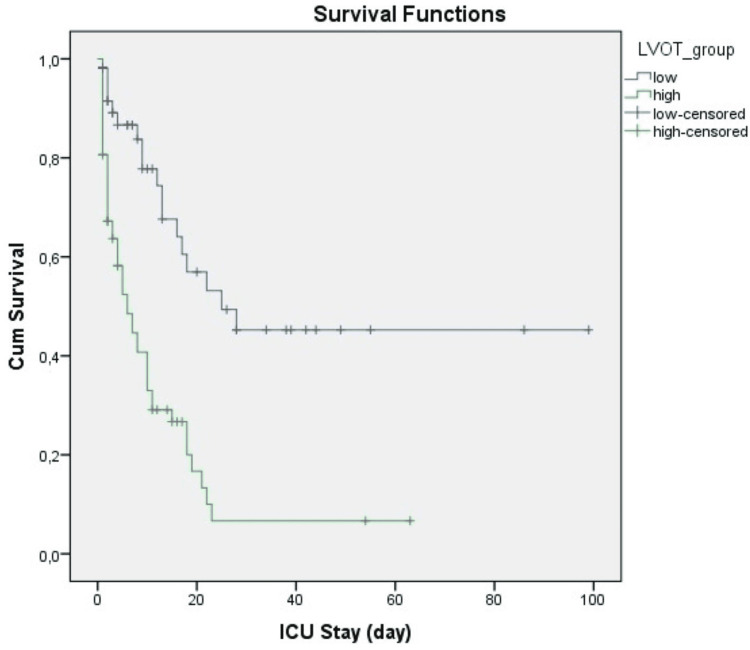
Kaplan-Meier analysis showing the 28-day mortality between high and low peak velocity groups

**Figure 2:**
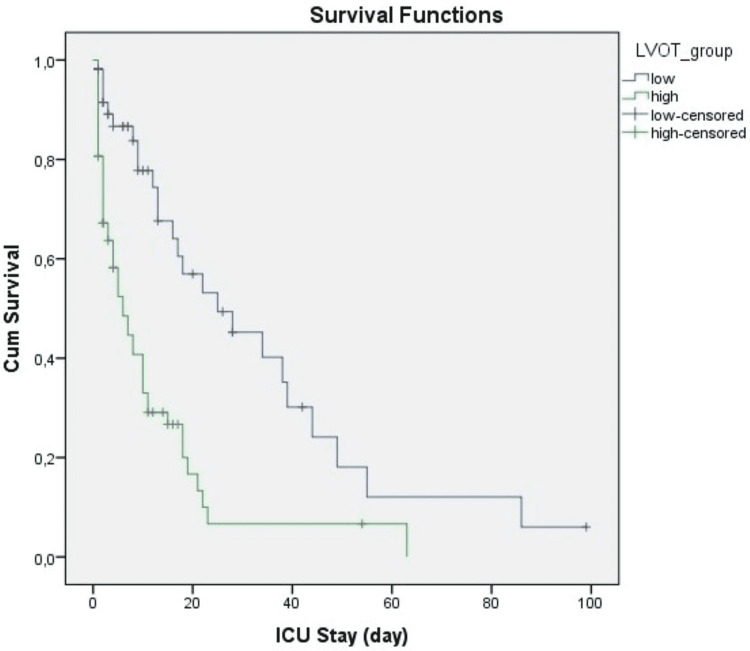
Kaplan-Meier analysis showing the in-hospital mortality between high and low peak velocity groups

The mean LVOT peak velocity in sepsis and septic shock groups was 71.5 ± 16.4 cm/s, and 87.3 ± 18.3 cm/s, respectively (p<0,001). A ROC curve analysis was performed to determine the efficacy of LVOT peak velocity measurement for discriminating septic shock from sepsis and predicting 28th day and in-hospital mortality (Table 2), and 75 cm/s was determined as a cut-off value as it corresponded with the highest Youden index. We re-grouped patients as low or high according to the LVOT peak velocity cut-off threshold and assessed any relationship between demographic and clinical parameters regarding low and high LVOT peak velocity groups (Table 3). Kaplan-Meier survival analysis showed statistically significant differences between groups with low and high LVOT peak velocity in both day 28 and in-hospital ICU death rates (Figure 1 and Figure 2).

**Table 2:** The diagnostic performance of LVOT peak velocity measurement (cut-off value is ≥75 cm/s) +LR: Positive likelihood ratio, -LR: Negative likelihood ratio, PPV: Positive predictive value, NPV: Negative predictive value

	Sensitivity (%)	Specificity (%)	+ LR	-LR	PPV (%)	NPV (%)	Accuracy (%)	p-value
Distinguishing sepsis from septic shock	79.3	63.8	2.2	0.3	68.7	75.5	71.5	<0.001
28-day mortality	78.5	68.6	2.5	0.3	76.1	71.4	74.1	<0.001
In-hospital mortality	71.2	65.1	2.04	0.4	77.6	57.1	68.9	<0.001

**Table 3:** Demographic and clinical features according to LVOT peak velocity measurement groups *According to the bedside measurements of inferior vena cava with ultrasound BP: Blood pressure, MAP: Mean arterial pressure, SOFA: Sequential organ failure assessment, SAPS: Simplified acute physiology score, MV: Mechanical ventilation, ICU: Intensive care unit, mRS: Modified Rankin scale

	Low Velocity (n=54)	High Velocity (n=62)	p-value
Age (years), Median (min-max)	74 (27-93)	71.5 (40-96)	0.857
Male, n (%)	34 (63)	34 (54.8)	0.376
Presence of Comorbidities, n (%)	42 (77.8)	51 (82.3)	0.546
Heart rate, (bpm) ± SD	105.4±21.5	104.6±20.3	0.262
Systolic BP (mmHg) ± SD	104.6±22.5	91.8±23.2	0.03
Diastolic BP (mmHg) ± SD	63.5±14.1	54.4±11.7	<0.001
MAP (mmHg) ± SD	76.1±15.0	66.8±15.2	0.01
Lactate (mmol/L), median (min-max)	3.4 (0.6-26)	3.1 (0.5-19)	0.612
SOFA, median (min-max)	6 (2-14)	8 (2-14)	0.005
SAPS 2, median (min-max)	41.5 (15-69)	49.5 (13-76)	0.01
Presence of hypovolemia*, n (%)	29 (53.7)	39 (62.9)	0.316
MV support, n (%)	11 (20.4)	19 (30.6)	0.207
ICU stay (days) median (min-max)	9 (1-99)	4 (1-63)	0.033
28-day ICU mortality , n (%)	18 (33.3)	47 (75.8)	<0.0001
In-hospital mortality, n (%)	25 (46.3)	48 (77.4)	<0.001
28-day mRS , median (min-max)	4 (1-6)	6 (3-6)	<0.001

## Discussion

In our study, when the LVOT peak velocity measurement was examined in the sepsis and septic shock groups, a significant increase was observed in the septic shock group compared to the sepsis group (p<0,001). In the ROC analysis performed to determine the power of LVOT peak velocity measurement in predicting septic shock, when the cut-off value was 75 cm/s, the desired sensitivity and specificity value was reached.

In a recent study with 40 patients with septic shock, the LVOT peak velocity measurement was observed to be significantly decreased following bolus fluid infusion at a rate of 30 cc/kg, and the presence of LVOT obstruction in a patient with septic shock suggested severe hypovolemia [7]. In our study, we found no significant difference between the LVOT peak velocities between patients with and without fluid deficit according to bedside caval measurements. Our study differs from the literature in this respect. We think that the reason for this situation may be due to patients with conditions such as right heart failure, pulmonary hypertension, and tricuspid valve insufficiency that may affect IVC measurements that are not excluded from the study.

In a case series published by Auer et al. in 2005, the authors suggested that hypovolemia and hyperdynamic conditions caused by catecholamine administration may result in LVOT obstruction, and discontinuation of catecholamine treatment can reverse this situation [8]. Another case series consisting of two cases published by Mingo et al. in 2006, showed that it is possible to see LVOT obstruction in patients on catecholamines and those having hemodynamic disorders [9]. In 2008, Yang et al. stated that the hemodynamic deterioration leading to LVOT obstruction is a side effect of catecholamine use under hypovolemia, and the obstruction will disappear with the discontinuation of catecholamine treatment and correction of hypovolemia [10]. On the other hand, in a study in 2015, Chauvet et al. compared patients with septic shock with and without LVOT obstruction [6]. All patients included in the study received at least one type of vasopressor/inotropic support. There was no significant difference between the groups with and without LVOT obstruction in terms of the type and dose of vasopressor/inotropic support. In our study, 96.5% of patients with septic shock received vasopressors (only noradrenaline). Although the aforementioned studies suggest that the use of catecholamines affects LVOT peak velocities, we assume that the effect of catecholamine infusion on LVOT peak velocity in our study would be minimal since very low doses of noradrenaline were being administered during the recruitment of the patients into the study.

In both the studies of Chauvet et al. and Elhadidy et al., the overall and 28th-day mortality rates were higher in patients with increased LVOT peak velocity [6,7]. Also, 90 cm/s was the cut-off value in both studies. Similarly, overall and 28th-day ICU mortality rates were significantly higher in the high peak velocity group of patients. We assume that LVOT peak velocity measurement may predict overall and 28th-day ICU mortality. However, our threshold was lower than the studies in the literature.

Regarding the length of stay of the patients in the ICU, we found no difference between the sepsis and septic shock groups. However, when patients were regrouped according to the LVOT peak velocity, the length of stay was significantly shorter in the high peak velocity group. Our study differs from the literature in this respect [6,7]. We think that the reason for this situation may be the shorter length of stay in the ICU due to the higher mortality rate in patients with higher LVOT peak velocity.

Our study has several limitations. First, ultrasonographic measurements were performed by only one physician. Therefore, consecutive measurements by a different physician could not be achieved and we were unable to perform correlation analyses including intra-class and inter-class correlations. Second, all measurements were made at the time of the ICU admission of the patients and were not repeated following fluid resuscitation.

## Conclusions

The LVOT peak velocity measurement yields different levels in sepsis and septic shock. We also found that higher LVOT peak velocity measurement may predict both 28th-day and in-hospital mortality. According to the logistic regression analysis, vasopressor administration did not significantly affect LVOT peak velocity measurements as well as the patients’ volume status. Therefore, we believe that LVOT peak velocity measurement may be a useful tool in the early management of septic patients in the emergency department, and critical care unit.
